# Preimplantation genetic testing for cardiomyopathies: a case series illustrating the clinical and technological perspective

**DOI:** 10.1093/ehjcr/ytaf630

**Published:** 2025-12-02

**Authors:** Isa M E Faassen, Malou Heijligers, Nadine H N Weijermans, Marianne L van Buul-van Zwet, Carlijn S Zietse, Masoud Zamani Esteki, Aimee D C Paulussen, Job A J Verdonschot

**Affiliations:** Department of Cardiology, Maastricht University, Cardiovascular Research Institute Maastricht (CARIM), Universiteitssingel 50, Maastricht 6229 ER, The Netherlands; Department of Clinical Genetics, Maastricht University Medical Center, P. Debyelaan 25, Maastricht 6229 HX, The Netherlands; Department of Obstetrics and Gynecology, Maastricht University Medical Center, P. Debyelaan 25, Maastricht 6229 HX, The Netherlands; Department of Obstetrics and Gynecology, University Medical Center Utrecht, Heidelberglaan 100, Utrecht 3584 CX, The Netherlands; Department of Obstetrics and Gynecology, University Medical Center Amsterdam, Meibergdreef 9, Amsterdam 1105 AZ, The Netherlands; Department of Clinical Genetics, Maastricht University Medical Center, P. Debyelaan 25, Maastricht 6229 HX, The Netherlands; GROW School for Oncology and Reproduction, Maastricht University, Universiteitssingel 40, Maastricht 6229 ER, The Netherlands; Department of Clinical Genetics, Maastricht University Medical Center, P. Debyelaan 25, Maastricht 6229 HX, The Netherlands; GROW School for Oncology and Reproduction, Maastricht University, Universiteitssingel 40, Maastricht 6229 ER, The Netherlands; Department of Cardiology, Maastricht University, Cardiovascular Research Institute Maastricht (CARIM), Universiteitssingel 50, Maastricht 6229 ER, The Netherlands; Department of Clinical Genetics, Maastricht University Medical Center, P. Debyelaan 25, Maastricht 6229 HX, The Netherlands; European Reference Network for Rare, Low Prevalence and Complex Diseases of the Heart (ERN GUARD-Heart), University Medical Center AMC, Meibergdreef 9, Amsterdam 1105 AZ, The Netherlands

**Keywords:** PGT, Cardiogenetics, Reproductive medicine, Case series

## Abstract

**Background:**

Preimplantation genetic testing (PGT) is increasingly used in patients with inherited cardiac disease. Technological advances have expanded its applicability, yet the process remains highly individualized and complex.

**Case summary:**

We present a case series of four patients undergoing PGT for various cardiogenetic disorders. The cases illustrate technical developments evolving from polymerase chain reaction (PCR) to whole genome sequencing (WGS). Each case highlights distinct aspects of the PGT process to illustrate the steps: (i) the variability in disease penetrance, (ii) the clinical decision-making process, (iii) the duration and timeline of the trajectory, and (iv) the sequencing methodology.

**Discussion:**

These cases emphasize the importance of selecting patients who will benefit the greatest risk reduction from PGT, reflecting the significance of individual assessment per couple. Additionally, clinicians should be aware and discuss the possibility of PGT with eligible patients consistently to ensure a comprehensive understanding of their reproductive options.

Learning points

- Clinicians should know how to select patients who will benefit the greatest risk reduction from preimplantation genetic testing (PGT), balancing clinical severity, genetic risk, technical feasibility, and psychological readiness.
- Clinicians should consistently discuss the option of PGT with eligible patients to ensure they fully understand the available reproductive options, thereby having the basic knowledge of a PGT procedure themselves.

## Introduction

**Figure 1:**
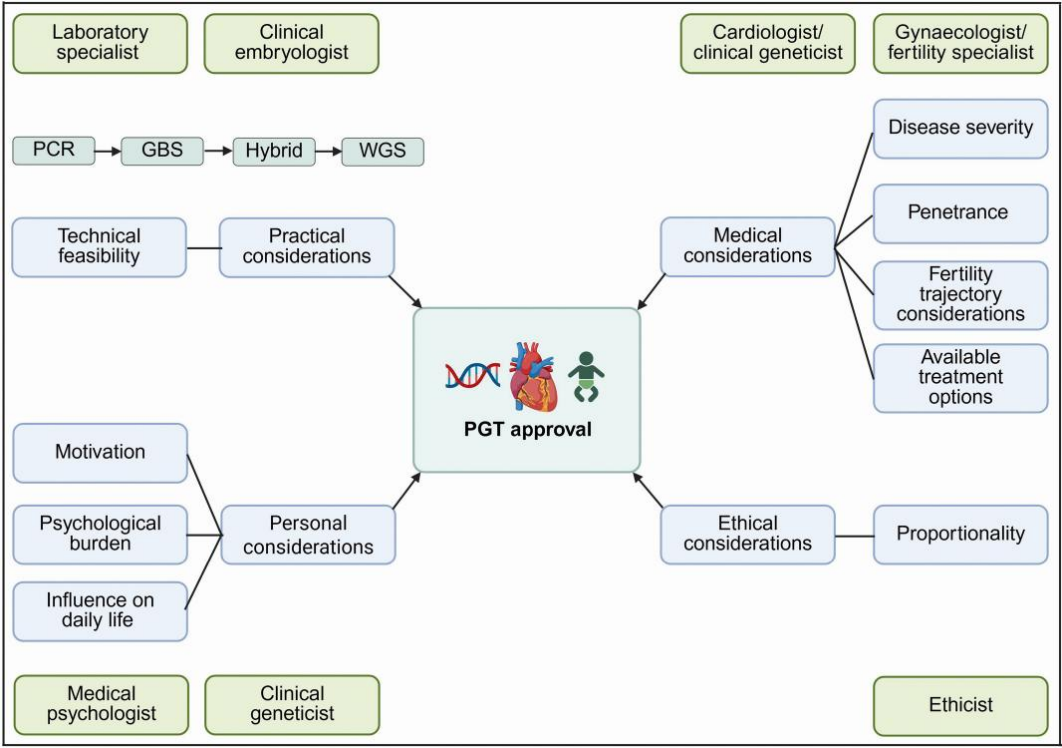
Determinants in the decision-making process of preimplantation genetic testing approval for inherited cardiac diseases. A multidisciplinary approach is used to determine which couples will have the greatest risk reduction from preimplantation genetic testing. Practical, personal, medical, and ethical aspects are considered. Ethical considerations involve assessing the principle of proportionality, evaluating whether the anticipated benefit of a preimplantation genetic testing trajectory outweighs the potential harms. GBS, genotyping by sequencing; WGS, whole genome sequencing; PCR, polymerase chain reaction; PGT, preimplantation genetic testing. This figure has been created in https://BioRender.com with the appropriate licence.

Preimplantation genetic testing (PGT) is a reproductive option that is receiving increasing attention by patients with an inherited cardiac disease.^1^ Preimplantation genetic testing for monogenic disorders (PGT-M) is designed to prevent transmission of a genetic disease to offspring by testing embryos on the familial pathogenic variant after *in vitro* fertilization (IVF).^2^ Although the allowed indications for PGT-M can differ per country, general consensus is that the genetic diseases typically must be early onset and cause a severe clinical phenotype with high penetrance to obtain sufficient risk reduction. However, the onset, disease severity, and penetrance of inherited cardiac diseases are highly variable, indicating that not everyone with the variant will develop a phenotype and if so, not at a similar age or severity. Preimplantation genetic testing for inherited cardiac diseases is therefore not straightforward. For example, until March 2025, all cardiogenetic indications for PGT were a ‘no, unless indication’. Proportionality in this context is applied to examine whether the probability of procreation with an unaffected child is weighted more heavily than the ethical concerns and the anticipated severity of the clinical trajectory. To assess this, individual cases are discussed in a multidisciplinary meeting and cardiologists are consulted on a case-by-case basis. Furthermore, all couples are scheduled for a session with a medical psychologist as part of our counselling process (*Figure 1*). A risk assessment model has been proposed to guide clinicians in the decision-making process for individual couples from a clinical perspective.^3^ The increasing knowledge of the genetic background of inherited cardiac diseases, combined with the broader implementation of genetic diagnostics in patients, leads to an increasing patient population that could benefit from PGT. Technological advancements in the genetic tests for PGT have created a shift from family-specific short tandem repeat(STR)–polymerase chain reaction (PCR) marker tests^2^ to next-generation sequencing, now providing the possibility to use generic whole genome sequencing (WGS)–based haplarithmisis.^4^ These technological advancements have made it possible to use generic tests for the majority of PGT couples and provide the option to include screening for other aberrations or information such as aneuploidies or sex chromosomes.^5^ The IVF procedure yields an average 20%–30% success rate for a successful pregnancy after an embryo transfer at our institute. In the current case series, we present four PGT-M cases with a variety of genetic variants and PGT techniques to provide a concise historical overview of the technological advancements and to illustrate the steps of a PGT process with real-life cases. All couples with an inherited cardiac disease are referred for counselling to the clinical genetics department of a designated university hospital within our country. After approval, they undergo the IVF procedure in one of the participating centres. While the overall procedure is fundamentally the same for all couples, each case highlights specific aspects of the PGT process. This does not imply that these aspects are not relevant to the other cases.

### Patient 1

**Figure 2:**
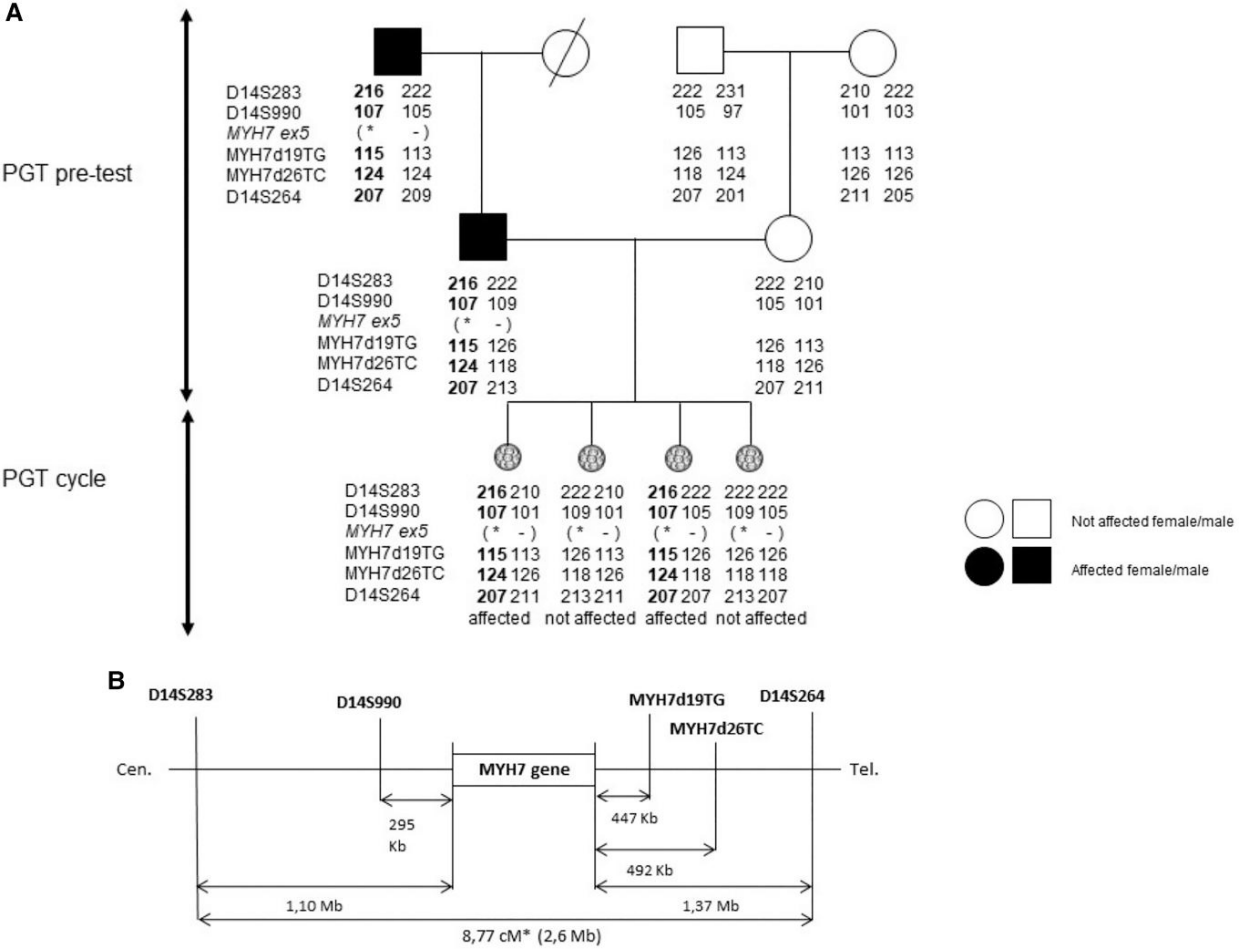
Haplotype phasing for genetic testing of embryos for preimplantation genetic testing for monogenic disorders using short tandem repeat-polymerase chain reaction set-up. (*A*) Pedigree of case one and haplotype phasing using short tandem repeat markers was employed to develop the pre-test. (*B*) To accurately distinguish between risk (bold) and non-risk haplotypes, multiple markers located at both centromeric and telomeric sites of the MYH7 gene (physical distance −1.10 Mb to +1.37 Mb) were analysed across two generations of family members. Five informative and semi-informative markers were identified. Cen., centromere; MYH7, myosin heavy chain 7; PGT, preimplantation genetic testing; Tel., telomere.

A 38-year-old male diagnosed with non-compaction cardiomyopathy (NCCM) around the age of 30 has a pathogenic *MYH7* variant (c.495G>A, p. Met165Ile) and opted for PGT-M. Segregation analyses of this variant in family members identified multiple carriers with NCCM. The PGT request was approved in 2018 after reclassification of the variant to pathogenic (Class 3 to Class 5) and the birth of their son with a NCCM due to the *MYH7* variant. Also his father, brother, sister, and two of his sister’s daughters have a NCCM phenotype and carry the *MYH7* variant. One of his sister’s daughters underwent a heart transplantation at a young age. Another sister of the proband died at the age of six due to a cardiomyopathy. Retrospective application of the risk prediction model yielded a score ≥ 10, indicating high disease expression and penetrance of the *MYH7* variant in this family.^3^ After approval, a PGT set-up protocol was developed using a multiplex STR-PCR protocol initially created for another family with a *MYH7* variant. As a result, the PGT set-up was completed within 2 months (*Figure 2*), substantially faster than the typical 12–18 months necessary for STR-PCR protocols. DNA of the parents of the partner and from the carrier father (reference for the risk-haplotype) was used to develop the pre-test (*Figure 2*). The test contained five STR markers flanking the MYH7 gene, two on the centromeric site and three at the telomeric site to determine the (bold) risk and non-risk haplotypes and control for allelic drop-out and possible recombination around the MYH7 locus. The first IVF cycle (June 2020) provided two unaffected, four affected, one with monosomy, and one inconclusive embryo. A second cycle (January 2021) yielded five unaffected embryos, three affected, and three with chromosomal abnormalities. Despite multiple embryo transfers, there was no successful pregnancy. The couple subsequently conceived spontaneously in 2021; the child carries the *MYH7* variant and is symptomatic. During pregnancy, an advanced foetal anomaly scan was performed. Additional invasive prenatal diagnostics were discussed, but not undertaken since pregnancy termination was not an option for the couple. The couple returned for a third cycle in May 2024, which yielded five unaffected and three non-conclusive embryos. The PGT trajectory is ongoing.

### Patient 2

A 26-year-old male with an arrhythmogenic right ventricular cardiomyopathy (ARVC) since the age of 17 due to a phospholamban (*PLN*) variant (c.40_42del, p. Arg14del), opted for PGT-M. The father and sister of the proband both carry the variant, but do not show a phenotype or symptoms. The sister of the proband’s paternal grandmother, also carrying the *PLN* variant, underwent a heart transplantation due to heart failure.

**Figure 3:**
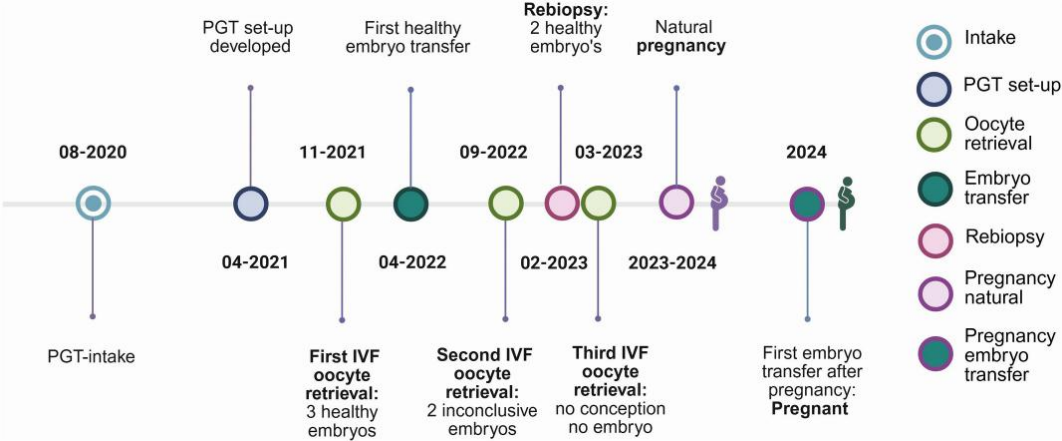
Timeline preimplantation genetic testing trajectory 2020–24. Preimplantation genetic testing trajectory from initial consultation to pregnancy after embryo transfer. PGT, preimplantation genetic testing. This figure has been created in https://BioRender.com with the appropriate licence.

The couple presented in 2020, and the multidisciplinary team approved the indication for PGT. Retrospectively, the decision model would have recommended approval based on variant classification.^3^ A PGT set-up was developed with a genotyping by sequencing (GBS) PGT. For the set-up, DNA from the couple and the parents of the proband was used. Instead of a low number of highly informative STR markers, the GBS method makes use of a high number of informative SNPs from a reduced genome to create genome-wide haplotypes.^6,7^ This generic method can be used for all PGT couples with a familial pathogenic variant. After generating reduced genome genotyping data, the bio-informatics data analysis pipeline can zoom in on the specific region of interest (ROI) flanking the familial variant/gene of interest in each PGT couple. As such, this method is less time-consuming and more cost-effective (no individualized set-up needed) (*Figure 3*). Checking all the required quality criteria with this method for the couple took five and half months and was finished in April 2021. The first IVF cycle (November 2021) produced three unaffected embryos. The first transfer did not result in pregnancy; the two remaining embryos did not survive thawing. A second cycle (September 2022) yielded two inconclusive embryos, later confirmed as unaffected via re-biopsy in February 2023. A third cycle (March 2023) did not yield viable embryos. Meanwhile, the couple conceived spontaneously in 2023, resulting in an unaffected child. In 2024, the first transfer of one of the re-biopsied embryos led to a second, ongoing pregnancy.

### Patient 3

**Figure 4:**
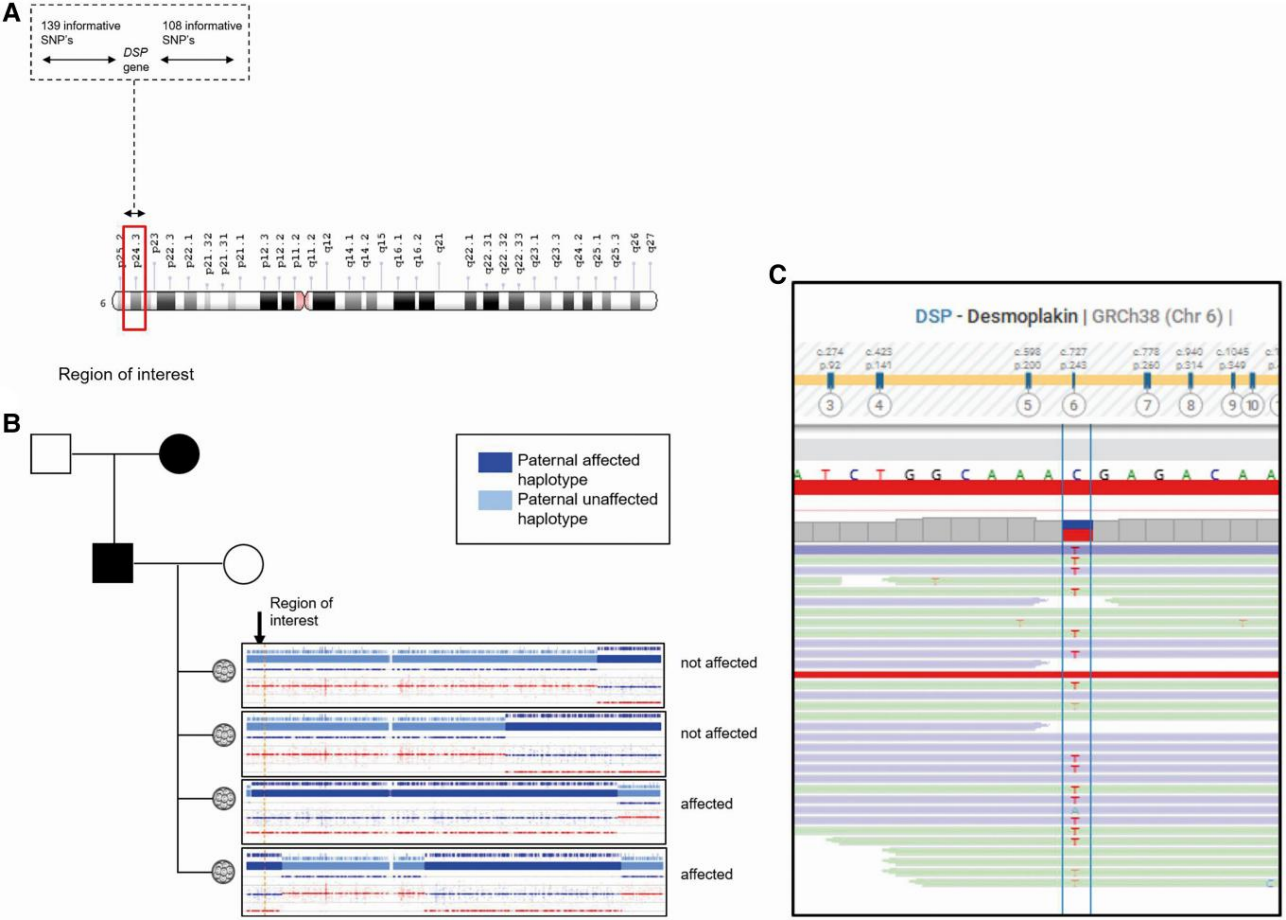
Preimplantation genetic testing set-up with genome-wide single nucleotide polymorphism haplotyping. The pre-test set-up was done with genotyping-by-sequencing preimplantation genetic testing and the embryo biopsies with whole genome sequencing preimplantation genetic testing . (*A*) Region of interest which includes the *DSP* gene. This picture shows a 2 Mb region proximal and distal of the *DSP* variant, with 139 informative single nucleotide polymorphisms on the left and 108 on the right side, using genotyping-by-sequencing preimplantation genetic testing. (*B*) The orange dashed line indicates the location of the gene to determine whether the embryo contains the paternal affected haplotype (dark blue) or unaffected haplotype (light blue) haplotype in the region of interest (the *DSP* gene). The first and the second embryo are unaffected. The results also show the high prevalence of chromosomal cross-over occurring in embryos. (*C*) Using whole genome sequencing, the exact single nucleotide variant can be visualized in the genetic data of the embryo biopsy. This specific alteration C>T displays an affected embryo. SNP, single nucleotide polymorphisms.

A 30-year-old male carrier of a desmoplakin variant (*DSP)* (c.2821C>T; p. (Arg941*)), without phenotype opted for PGT. The male carrier has currently no signs or symptoms of the disease and remains under cardiac screening once every 2 years. His mother has a cardiomyopathy and the daughter of a sister of mother’s father had an indication for a heart transplantation around the age of 60 years. Additionally, mother’s sister exhibits a dilated cardiomyopathy phenotype, and her daughter is carrier of the DSP variant. The indication for PGT was approved by the multidisciplinary team, which considers not only genotype but also phenotype severity, psychological burden, technical feasibility, and ethical aspects (*Figure 1*). Although the patient had no clinical symptoms, the cumulative factors justified proceeding with PGT. A hybrid approach was used for PGT: GBS for the test set-up and WGS on embryo biopsies (*Figure 4*).^5,6^ The first PGT cycle obtained two embryos, of which one was affected (March 2023). The unaffected embryo was transferred (June 2023), resulting in a biochemical pregnancy. A biochemical pregnancy is an early pregnancy loss that occurs shortly after implantation. The second cycle (November 2023) produced 10 embryos (4 unaffected, 6 affected); none of the transfers led to pregnancy. The third cycle (November 2024) yielded five unaffected and four affected embryos, with the first transfer resulting in a successful pregnancy.

### Patient 4

**Figure 5:**
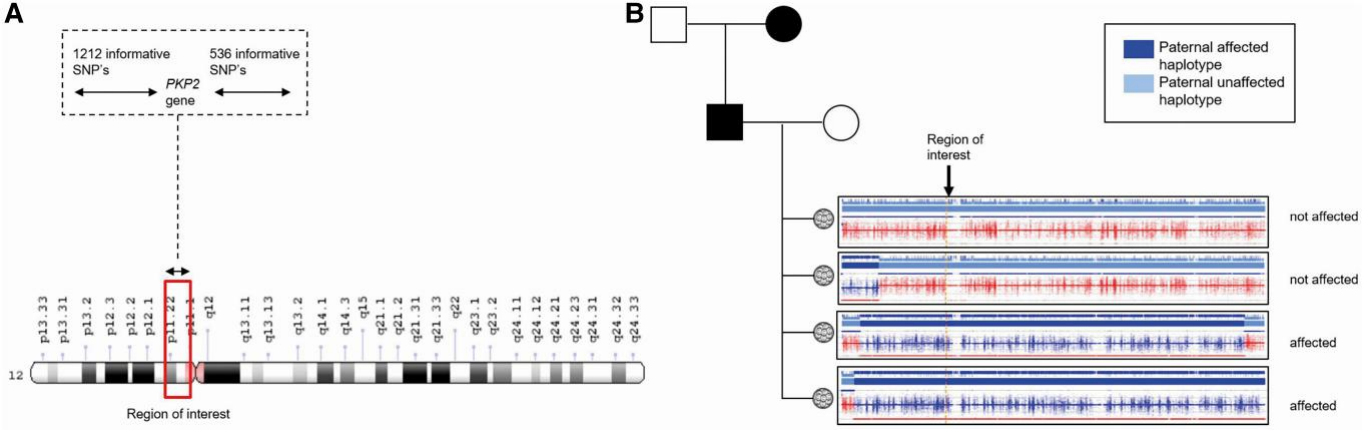
Preimplantation genetic testing set-up with whole genome-wide single nucleotide polymorphism haplotyping in pre-test and embryo biopsy. (*A*) Region of interest containing the *PKP2* gene. The figure illustrates a 2 Mb region flanking the genetic variation, with 1212 informative single nucleotide polymorphisms proximal and 536 distal of the gene, whole genome sequencing. The number of informative single nucleotide polymorphisms is substantially higher compared to reduced preimplantation genetic testing (*Figure 4*). (*B*) Using informative single nucleotide polymorphisms, the paternal haplotype at the region of interest can be determined from every embryo. Both the first and second embryos have the paternal unaffected haplotype. PKP2, plakophilin 2; SNP, single nucleotide polymorphisms. This figure has been created in https://BioRender.com with the appropriate licence.

A 25-year-old male with an ARVC due to a pathogenic *PKP2* variant (c. 1689-?_2646+?del) opted for PGT-M. This patient was diagnosed at age 16 following a cardiac arrest, after which an implantable cardioverter-defibrillator (ICD) was placed. Over the years, the ICD delivered multiple appropriate shocks. His mother, sister, and grandmother also have the same variant in *PKP2*, although none of them exhibit clinical symptoms. Retrospectively, the risk assessment model would have advised approval.^3^ Whole genome sequencing was used for both the pre-test set-up and embryo biopsy analysis, completed within 6 months. Compared to traditional STR marker-based PCR methods, WGS via SNP analysis and standardized protocols offers a faster and more accurate test set-up. Genotyping by sequencing (applied in Patients 2 and 3) provides similar accuracy but targets only the gene ROI, reducing cost while losing genome-wide resolution.^8^ Whole genome sequencing remains more informative overall (*Figures 5* vs. *4*) and, due to decreasing costs, is now the standard for PGT. The first round of IVF yielded six unaffected, five affected, and four inconclusive embryos (January 2024). The first embryo transfer (April 2024) resulted in a successful pregnancy. The full trajectory (from genetic intake at our department to successful pregnancy) went without complications, still spanned 15 months.

## Discussion

These cases illustrate key clinical and technological aspects in the dynamic field of PGT. As our knowledge on the genetics of inherited cardiovascular diseases increased over the years, PGT has become an important option for patients. While PGT provides an interesting reproductive possibility for patients, the variable disease penetrance complicates decision-making for patient and counsellor. The European Society of Cardiology and European Heart Rhythm association acknowledge the absence of strategy guidelines for cardiogenetic indications.^3^ In the family of Patient 1, the *MYH7* variant showed high penetrance, in contrast to the other families with multiple unaffected carriers. Patient 4 has a severe phenotype associated with a *PKP2* variant, suggesting additional genetic modifiers. Performing PGT for the *PKP2* variant might remove an important genetic aetiology, but there might still be an unknown residual risk for phenotype development, which is important in the counselling process.

Importantly, PGT is also an option for unaffected carriers, as in Patient 3. Risk models can aid in estimating benefit, based on family history and disease expression.^3^ In this case, the severe disease expression in the family was a strong medical argument to argue for allowing PGT. The expanding application and possibilities of PGT in cardiovascular diseases highlight the importance of informing and discussing the possibility with every patient. Current technological advancements, particularly the shift from PCR-based STR markers to WGS, have improved the efficiency and accuracy of PGT, reducing inconclusive results and enabling precise breakpoint detection. However, it should be noted that the availability of WGS is restricted and not yet implemented worldwide. Germline genome editing might be a future technical possibility to increase the number of embryos that can be transferred, but ethical concerns and public resistance currently restrict its acceptance.^9,10^ Additionally, the process of PGT remains time-intensive (e.g. in some cases multiple years) and emotionally demanding without guarantee of a successful pregnancy. *In vitro* fertilization may yield limited viable embryos, and pregnancy rates per embryo transfer remain around 20%–30%. In general, PGT itself has no effect on the success rate of IVF treatment, although the success rate is usually higher for PGT couples as there is no underlying fertility problem.^11^ Careful selection of candidates is crucial, balancing clinical severity, genetic risk, technical feasibility, and psychological readiness to select patients who will benefit the greatest risk reduction from PGT. As the indications and capabilities of PGT expand, it is essential that clinicians consistently discuss this option with eligible patients and families.

## Patient’s perspective

The couple (patient 4) described the PGT trajectory as a unique opportunity. The waiting and the IVF trajectory are mentally and physically challenging, for which a team with expertise is essential. The couple was grateful that they were able to have a healthy child of their own.

## Lead author biography


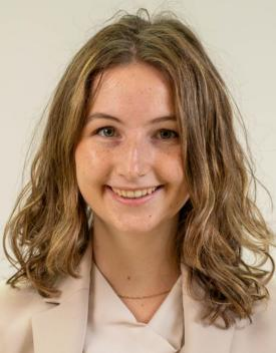


Isa M.E. Faassen, MD, is a cardiology research physician at Maastricht University. Her clinical and research interests focus on genetic heart disease, with a particular emphasis on preimplantation genetic testing and incidental findings. In the future, she aspires to pursue a career in clinical genetics.

## Data Availability

The data underlying this article will be shared on reasonable request to the corresponding author.
